# Fundamentals of burrowing in soft animals and robots

**DOI:** 10.3389/frobt.2023.1057876

**Published:** 2023-01-30

**Authors:** Kelly M. Dorgan, Kathryn A. Daltorio

**Affiliations:** ^1^ Dauphin Island Sea Lab, Dauphin Island, AL, United States; ^2^ School of Marine & Environmental Sciences, University of South Alabama, Mobile, AL, United States; ^3^ Mechanical Engineering Department, Case Western Reserve University, Cleveland, OH, United States

**Keywords:** soft robotics, sediments, soils, fracture mechanics, annelids

## Abstract

Creating burrows through natural soils and sediments is a problem that evolution has solved numerous times, yet burrowing locomotion is challenging for biomimetic robots. As for every type of locomotion, forward thrust must overcome resistance forces. In burrowing, these forces will depend on the sediment mechanical properties that can vary with grain size and packing density, water saturation, organic matter and depth. The burrower typically cannot change these environmental properties, but can employ common strategies to move through a range of sediments. Here we propose four challenges for burrowers to solve. First, the burrower has to **create space** in a solid substrate, overcoming resistance by e.g., excavation, fracture, compression, or fluidization. Second, the burrower needs to **locomote into the confined space**. A compliant body helps fit into the possibly irregular space, but reaching the new space requires non-rigid kinematics such as longitudinal extension through peristalsis, unbending, or eversion. Third, to generate the required thrust to overcome resistance, the burrower needs to **anchor within the burrow**. Anchoring can be achieved through anisotropic friction or radial expansion, or both. Fourth, the burrower must **sense and navigate** to adapt the burrow shape to avoid or access different parts of the environment. Our hope is that by breaking the complexity of burrowing into these component challenges, engineers will be better able to learn from biology, since animal performance tends to exceed that of their robotic counterparts. Since body size strongly affects space creation, scaling may be a limiting factor for burrowing robotics, which are typically built at larger scales. Small robots are becoming increasingly feasible, and larger robots with non-biologically-inspired anteriors (or that traverse pre-existing tunnels) can benefit from a deeper understanding of the breadth of biological solutions in current literature and to be explored by continued research.

## 1 Introduction

As the advancing field of soft robotics enables fabrication of soft, autonomous robots, a natural question arises: how can robots emulate the burrowing proficiency of the animals that are diverse and abundant in both terrestrial and marine environments? Many burrowing animals, especially those with soft bodies, have elongate, worm-like forms. Similarly, plant roots that grow by similar mechanisms are elongate. Interest in worm-like robots grows from both the apparent simplicity of biological worms and the application potential, including stabilization of marine instrumentation, non-disruptive soil and sediment exploration for agriculture or civil projects, and new approaches to laying cables in more cost-effective and less destructive ways. Live worms span orders of magnitude in size, from smaller than sediment grains to over a meter long, and vary in motility, burrowing depth, behaviors, and mechanisms of burrowing, and thus provide a rich set of examples for bioinspired design.

One of the key challenges in bioinspired design is to identify the characteristics of the organism that are most critical to the design function (Kovač, 2014; Mazzolai and Laschi, 2020). Kovač (2014) describes an “inspire - abstract - implement (IAI)” design flow, in which the underlying design principles in biology are identified (abstracted) and then implemented using state-of-the-art technology. Ideally, these fundamental principles of locomotion are captured in a simple model. For walking, for example, a simple inverted pendulum serves as a template, capturing the exchange between potential energy and kinetic energy as the center of mass changes height (Full and Koditschek, 1999). This template model is general enough that it relates to many different animals and robots with walking gaits. Individual organism models that are more detailed and complex serve as anchors within the template (Full and Koditschek, 1999). Models enable more effective application of bioinspired design by identifying the most general principles of the biological system rather than replicating the detailed forms of the organisms (Full and Koditschek, 1999; Martinez et al., 2021).

Here we aim to describe general patterns in the mechanics of burrowing across diverse burrowing animals and identify the underlying principles that are most relevant to development of bioinspired burrowing robots. We emphasize soft-bodied burrowers as inspiration for the rapidly growing field of soft robotics (see reviews in (Lachi et al., 2016; Whitesides 2018; Mazzolai et al., 2022)), while drawing examples from a broader diversity of burrowers to generalize the underlying principles. We also address how environmental complexity, specifically soil or sediment properties, affects burrowing mechanics, and highlight body size as an important parameter affecting burrowers (Dorgan, 2015).

In addition to addressing new robotic applications, bioinspired robots are potential tools for studying biology. Burrowers are difficult to observe in their natural environments due to the opacity of soils and sediments, and transparent analogs for soils and sediments (e.g., gelatin) have numerous limitations (Dorgan et al., 2005). Burrowing animals need not only to move through their environment, but they also need to consume, digest, and egest food, obtain oxygen, avoid predators, and reproduce. Understanding the fundamental principles of burrowing allows us to distinguish among these different functions. Additionally, better understanding of the fundamental principles in burrowing provides insight into the evolution of body forms and behaviors of the diverse animal communities living in marine sediments.

## 2 Fundamental problem of burrowing

We define burrowing as locomotion through substrate that is solid but soft enough to be compressed to create space (Dorgan, 2015). In contrast, “boring” is the mechanical or chemical excavation of a material that is too stiff to be compressed (e.g. in tunnel boring machines (Maidl et al., 2008)), and “swimming” is locomotion through a substrate that is fluidized and flows rather than deforms in response to the forces applied (e.g. in sandfish swimming (Maladen et al., 2009)). Because the mechanical properties of these media are different, the challenges of locomotion also differ (Dorgan, 2010).

Burrowing in animals has been honed for power efficiency rather than long-distance travel. Burrowing first appears in the fossil record around the time of the Cambrian explosion, a rapid diversification of animal life (Meysman et al., 2006). Presumably, sediments were a refuge from predators, who shortly afterwards began burrowing in search of their hidden prey in an evolutionary “arms race” (Thayer, 1979). Because moving even a short distance in soil effectively hides prey, burrowing animals do not travel large distances. Most marine invertebrates that live in sediments have planktonic larvae that can be transported by currents over large distances. Burrowing is energetically costly per distance traveled when compared to flying, swimming or running (Dorgan et al., 2011). During the burrowing stages of their lifecycles, animals move slowly enough that the additional energy expenditure rate when burrowing is small compared to resting (Dorgan et al., 2011).

Diverse burrowing strategies have emerged for animals with a range of body sizes and environments (Table 1). For very small animals, burrowing may be limited only by the strength requirements to push a single sediment grain. We focus here on animals that are much larger than grain sizes, such that soils or sediments behave like a continuum. For large burrowers, e.g., mammals such as moles and rodents, the burrow diameters are limited by the strength of the soil needed to prevent collapse (Carotenuto et al., 2020). Responses of sediments to forces applied by burrowing animals depends on whether the sediment is cohesive or granular. Sands are granular materials that can be compacted or excavated or can temporarily behave like a liquid if grains are suspended in fluid so that individual grains do not rest on each other (Dorgan, 2015). Most of the sea floor is muddy, however, and muds deform elastically on small scales of burrowing animals because the fine particles are connected with organic material (Dorgan et al., 2006). Animals extend burrows through muds by fracture (Dorgan et al., 2005). Despite differences in substrate mechanics, burrowing animals show common patterns of movement. Burrowers apply forces to the walls of the burrow close to the tip; these normal forces propagate fractures in muds and reduce penetration resistance in granular materials (Dorgan, 2015; Martinez et al., 2021).

**TABLE 1:** Mechanisms to create burrows in different sediment types by animals of different body sizes.

	Sediment type		
Body size	Unconsolidated surface sediment	Cohesive, elastic muds	Non-cohesive granular sands
Diameter << 1 mm	Interstitial/compaction	Crawl within larger burrows	Interstitial crawling
Diameter ∼ mms, worms	compaction	Fracture to extend anteriorly	Compaction, radial expansion to reduce anterior stress
Diameter ∼ cm, fiddler crabs, large worms	compaction	Fracture with compaction and excavation	Some compaction, mostly excavation
Diameter > cm, crabs, mammals	compaction	Excavation, a little compaction for stabilization of burrow walls	Excavation at depth; fluidization near surface with no cohesion

**FIGURE 1:**
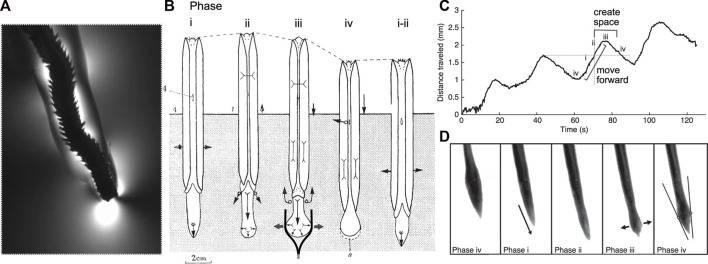
Burrowing by worms and clams uses similar mechanisms and phases of behaviors. **(A)** The polychaete *Alitta virens* extending a burrow by fracture in seawater gelatin (modified from Dorgan et al., 2007). Phases of burrowing cycles by **(A)** the razor clam, *Ensis* (Trueman 1968; Figure 4) and **(B,C)** the worm, *Cirriformia* (modified from Che and Dorgan 2010b; Figure 3) are consistent with this mechanism. **(B)** Schematic diagram of clam burrowing in sediment, showing alternating penetration anchor (phase i) and terminal anchor (phase iii-iv). (i) The shell expands to hold the body in place while the foot moves anteriorly, (ii) then the shell begins to close, expelling fluid from the mantle cavity (black arrows) as the foot begins to dilate. (iii) The foot expands to create a terminal anchor, applying force to the burrow walls (gray arrows) that in mud would extend the burrow by fracture (as drawn) and in sand would create a low-stress region in front of the foot. The shell is pulled forward, and additional fluid expelled from the mantle cavity reduces friction with the burrow wall. (iv) The foot is relaxed and pulled backwards and the shell begins to expand to repeat the cycle. (Phase numbers were changed to match those described by Che and Dorgan, 2010a). **(C)** Distance travelled by *Cirriformia moorei* in gelatin, showing cyclic movement of the anterior of the worm. **(D)** Images of *C. moorei* burrowing in gelatin, with phases corresponding to those indicated in the graph in **(C)** and the description of *Ensis* in **(B)**. Circles in phase iv indicate contact points of the worm with the burrow wall, and the lines show the tangent to those points (see Che and Dorgan 2010b for more detail).

Many burrowing animals are elongate worms that move by expanding and contracting segments or regions of the body. Different segments alternate between moving forward and anchoring, resulting in discrete steps of forward movement for each segment (Figure 1). When these sequential expansions and contractions occur in waves, this is called peristalsis (Gray and Lissmann, 1938). Engineering implementations of peristaltic locomotion provide insight on how animals and robots use these waves of muscular contraction to achieve forward movement. It has been shown that worm-like robots can be constructed from a wide range of materials and actuators, see Kandhari review of over 30 examples (Kandhari et al., 2021). Segment actuations must be coordinated to result in locomotion, however, and this coordination itself varies if the substrate is uneven (Daltorio et al., 2013), or if the robot is turning (Kandhari et al., 2019b). The simplest substrates for peristaltic movements are flat ground and smooth interiors of tubes. Burrowing through solid substrates using peristalsis has proven much more challenging for robots. However, Omori et al. (2013) use a combination of peristaltic locomotion and a rotating auger head to burrow in seabeds (Isaka et al., 2019; Okui et al., 2021). In this case, peristaltic locomotion was effective once a burrow was created with an alternate method. Some recent bioinspired robots demonstrate locomotion through shallow dry sand, but with mechanisms even more divergent from worms, such as terrafoils or flexible limb-like structures (Drotman et al., 2022). Tip extension by eversion reduces skin drag, and combined with air flow and a wedge tip, was successfully implemented in a robot that burrows in dry granular media, inspired by plant root growth, sand fluidization by burrowing octopus, and the asymmetrical wedge-shaped head of the sandfish lizard, respectively (Naclerio et al., 2021). Our goal in this paper is to compare these robots with live animals, in order to understand fundamental principles that can be applied to both.

**FIGURE 2:**
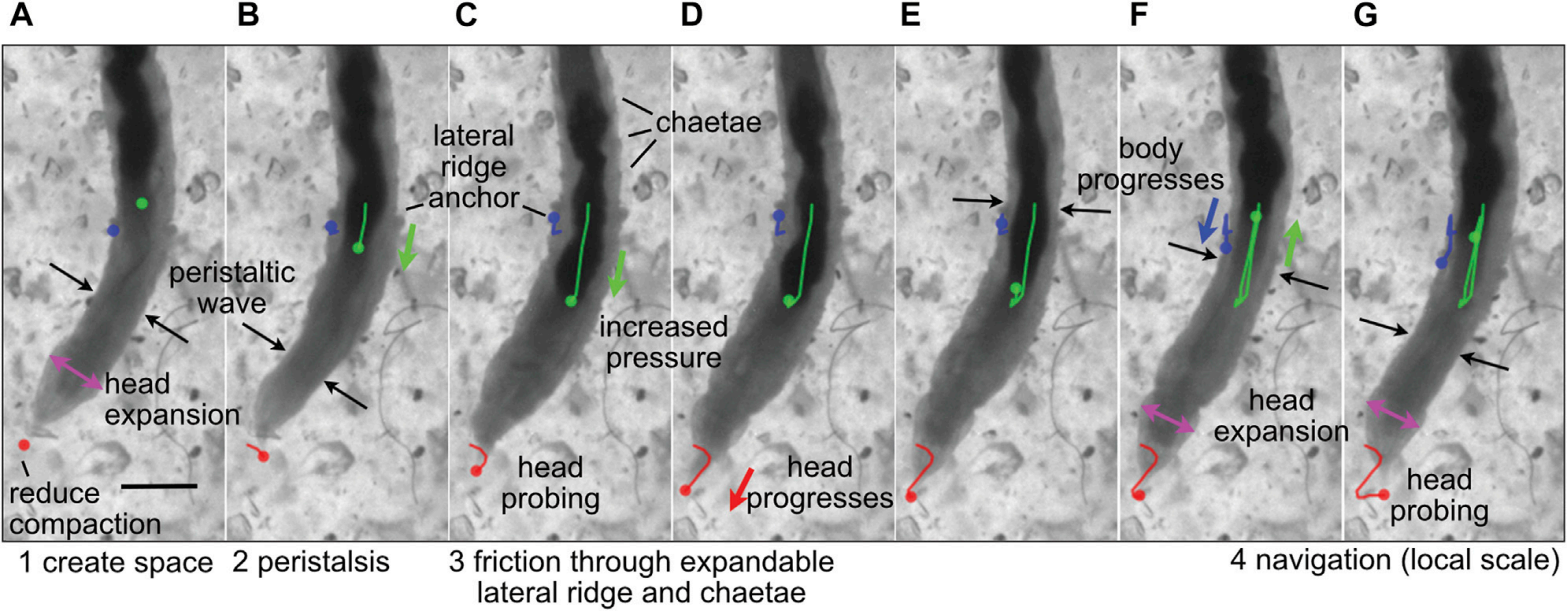
Image sequence of the polychaete worm, *Thoracophelia mucronata,* burrowing in cryolite grains, a transparent sand analog. **(A)** The worm expands its head (magenta arrows), reducing compaction of sand in front of the head (tip marked with red dot), as **(A,B)** a wave of contraction (black arrows) moves anteriorly in direct peristalsis. **(B–D)** The worm anchors its posterior region by expanding balloon-like appendages on both sides of the 11th segment, called lateral ridges (blue), and extending hairs called chaetae from other segments to increase friction **(C,D)** Segments posterior of the lateral ridge contract to push the gut (darker color, marked with green) forward, increasing pressure in the anterior region of the worm to push the head forward. The tip of the head probes **(C,G)** to find the easiest path forward through the sand grains. Colored dots show the location in the current frame of the tip of the head (red), anterior point in the lateral ridge (blue) and anterior point in the gut that can be seen through the body wall (green), and lines in **(B–G)** show the tracked progression of that position from image **(A)**. Modified from Figure 4 of Dorgan (2018) with permission.

We propose to distill burrowing into four essential tasks. Burrowers need to 1) make new space in the solid substratum to move forward. Next, they need to 2) advance the soft body forward into that space. To achieve and maintain this forward progress, they need to 3) anchor against the confined space of the burrow interior. These steps generally occur in discrete burrowing cycles in which parts of the animal alternate between anchoring and moving (Figures 1, 2). Finally, 4) the burrower needs sensory and navigational abilities, both locally to achieve effective movements and on longer scales to be able to control the direction of locomotion and navigate through the substrate. Our goal to consider approaches to burrowing from the perspective of the burrower and to integrate our understanding from biology and robotics distinguishes our review from previous reviews of burrowing. Wei et al. (2021) provide a valuable categorization of burrowing robots, especially those that could be used in planetary soils; Martinez et al. (2021) provide an overview from a soil mechanics perspective. Hosoi and Goldman review strategies of burrowing in granular media by size and speed regimes, providing canonical biological examples and mathematical models for their relevant soil mechanics (Hosoi and Goldman 2015). Dorgan (2015) also distinguished among sizes of burrowing animals in a review from a biological perspective.

Dividing burrowing into these components allows deliberate examination of how animals and robots use different structures and behaviors to solve these problems. For example, the worm, *Thoracophelia mucronata* (Figure 2), uses fundamentally different mechanics to create space to move forward from the animals in Figure 1, but moves forward in similar ways. The expansible lateral ridge and chaetae provide anchoring, and the alternating contraction of circular and longitudinal muscles create direct peristaltic waves for forward motion (Dorgan, 2018). Scaling differences and environmental constraints affect these problems differently, with animals using different strategies depending on body size, sediment type, and evolutionary history (Dorgan, 2015). Additionally, these problems may vary in their importance for different engineering applications, e.g., burrowing through harder substrata may require greater emphasis on creating space, whereas if precision in locating a target is important, then navigation may be a bigger challenge. We argue that solving these four problems should allow a robot to burrow effectively, i.e., to make forward progress through soils or sediments.

Live worms and robots do not necessarily solve these four challenges discretely, rather use strategies that integrate the solutions to multiple of these challenges. More complex behaviors will build on these templates for understanding burrowing animals and building burrowing robots. For example, the same sensory and control mechanisms that enable efficient locomotion can also be used to make decisions about avoiding obstacles, make observations of the environment, or improve designs over time. Energetic trade-offs among design considerations, e.g., soft-bodied *versus* hard-bodied burrowers or the number, shape and actuation of segments, can be investigated. Energetic efficiency will enable increasingly autonomous robots. For biologists, better understanding of burrowing effectiveness and efficiency will improve predictions of how environmental changes influence burrowing animals that play important roles in ocean ecosystems.

## 3 Burrowing challenge 1: Making space

### 3.1 Mechanics of soils and sediments

Making space in solid substrata requires an irreversible change in the sediment structure, or material failure to create a new surface and space within the solid. The mechanisms that animals use to extend burrows anteriorly depend strongly on both the properties of the soil or sediment and the size of the burrower (Dorgan, 2015). The mechanical properties of soils and sediments vary substantially, from coarse-grained granular sands to cohesive muds to fine-grained sediments that can have high enough porosities to behave like fluids (Dorgan et al., 2006). In granular sands, gravitational forces dominate, whereas muds have pore spaces that are filled with a matrix of organic matter (Watling, 1988), and the adhesion and cohesion of that organic material can be important in mechanical behavior (Dorgan et al., 2006). In terrestrial soils, water content can change over short time scales and distances, affecting the cohesion and compaction (Mitchell and Soga, 2005).

In sands, forces are transmitted along grain-grain contacts that form stress chains, which means that penetration resistance can vary on small scales depending on the distribution of overlying weight (Geng et al., 2001). Dry granular media behaves like a frictional fluid in response to burrowing, which has been modelled using a resistive force theory (RFT) that balances thrust and drag forces (Maladen et al., 2009; Maladen et al., 2011). Penetration resistance is greater in wet (unsaturated) than in dry granular media, when grains are packed more tightly, and when grain-grain friction is higher (Sharpe et al., 2015). This is illustrated by a comparison of worms burrowing in grains of cryolite, a transparent sand analog, *versus* in glass beads. Substantially more grain movement occurred around animals in glass beads than cryolite; lower grain-grain friction likely makes glass beads more easily fluidized (Francoeur and Dorgan, 2014). Although some worms are able to burrow as effectively in glass beads as in cryolite, others showed behavioral changes consistent with less penetration resistance and more backward slipping (Francoeur and Dorgan, 2014; Dorgan, 2018).

Muds can behave elastically and fail by fracture or be compacted and yield more plastically. Burrows are extended through muds by fracture (Dorgan et al., 2005). Burrows are tongue-depressor shaped, elongate disk-shaped cracks, and animals apply forces to the walls of the burrow that create tensile stress at the crack tip (Figure 1A). When enough stress is applied to exceed the fracture toughness, the burrow extends by fracture. These normal forces applied by worms against the elastic burrow walls result in stored elastic energy that is released during fracture to create new burrow surface area. Muds fail by fracture under tensile stresses, but can yield or plastically deform under compressive stresses. Compression presumably allows for formation of a cylindrical burrow from an initial crack, although the mechanisms have not been well studied. A challenge for both worms and robots in extending burrows by fracture is to apply forces large enough to overcome the fracture toughness but not so large that they over-expand the burrow.

### 3.2 Burrowing strategies vary with body size

Creating space becomes increasingly challenging with increasing burrower size, and animals of different sizes use different strategies and tools. The smallest animals are so much smaller than sediment grains that they move through existing spaces, without having to create space (Giere, 2009).

#### 3.2.1 Worms and other small burrowers

For small burrowers, e.g., worms that have diameters on the order of millimeters, the mechanics of burrow extension differs between granular sands and cohesive muds. Worms extend burrows through muds by fracture (Dorgan et al., 2005). In contrast, burrow extension in sands by worms of similar sizes is achieved by extended a narrow, pointed anterior into a space between grains, then expanding the head radially and compacting the sand (Dorgan, 2018) (Figure 2). In granular materials, body expansions can reduce the effective stress in front of the expansion, facilitating forward movement (Chen et al., 2021). Although mechanics differ, behaviors of the worms are similar, applying normal forces to burrow walls and minimizing friction between the body and the burrow walls (Dorgan, 2015).

Clam-inspired robots have shown that penetration strategies can be different for vertical burrowing up *versus* down. RoboClam, a biomimetic robot based on the razor clam, *Ensis directus*, expands the clam shell before moving forward, reducing penetration resistance (Winter et al., 2010). The mechanism for the observed reduction in energetic cost is likely crack propagation or reducing effective stress in a granular material. In live clams, downward burrowing is an order of magnitude slower and involves injection of water to fluidize sand. Unlike worms which can use similar strategies to burrow forward and backward (Che and Dorgan, 2010a), clams alternate expansions of a hard shell above and a soft foot below. SBOR, a simple razor clam inspired robot model, demonstrates that merely extending and retracting the body results in upward burrowing due to the flow of sand (Tao et al., 2020). Although behaviors of burrowing animals show similarities in muds and sands that have different mechanical responses, burrowing behaviors and performance of animals vary substantially across different burrowing substrates. Because sediments are opaque, numerous transparent analogs have been used to study burrowing behaviors. Methylcellulose mixed with seawater is a viscous fluid that has been used to study burrowing in very soft oozes (Hunter, 1982). However, most muds are elastic solids, and the animals that inhabit them have difficulty burrowing in methylcellulose, slipping backwards and failing to make forward progress (Dorgan, pers. obs.). Seawater gelatin has similar fracture properties to elastic muds and has been used as a transparent burrowing medium to study the mechanism of burrow extension (Dorgan et al., 2005; 2007). Worms successfully burrow in other transparent elastic gels, exhibiting burrowing behaviors that depend on the fracture toughness and stiffness of the gel (Dorgan et al., 2008). Gelatin differs from muddy sediments in two important ways: first, sediments exhibit hysteresis (the loss of stored elastic energy) and yielding, whereas gelatin does not yield or exhibit loss of stored energy under the forces applied by burrowing animals (Dorgan et al., 2007). Second, sediments are heterogeneous with material properties that vary on small scales relevant to burrowing animals (Watling, 1988); recent work shows that heterogeneity is important in crack branching and navigation by animals that extend burrows by fracture (Dorgan and Arwade, submitted). Thus, gelatin is an appropriate analog material for studying burrow extension by fracture, but is less appropriate for longer-term behavioral studies. Worms that use mechanisms other than fracture to extend burrows are generally unwilling or unable to burrow in gelatin or show reduced performance (Dorgan et al., 2013; Francoeur and Dorgan, 2014).

#### 3.2.2 Larger burrowers: Excavation and fluidization

Larger burrowers excavate burrows in deeper sediments by scraping the anterior burrow wall and transporting boluses of soil or sediment up to the surface. Most of the energetic expenditure to excavate burrows by wolf spiders is due to scraping and dislodging soil with their scraping fangs; the energetic cost of transporting the soil boluses was estimated to be <10% of dislodging soil (Suter et al., 2011). (Burrows extend ∼13 cm deep and have diameters of ∼1.5 cm) Mammals that burrow in terrestrial soils have claws for scraping and excavating soils. Burrow diameters (ranging from 3–15 cm) are constrained by the compressive strength of the soil the animals inhabit (Carotenuto et al., 2020). Carotenuto et al. (2020) suggest that compressive strength of soils limits the body size of these animals: larger animals have larger burrow diameters, which would collapse if the soil strength is too low. Animals such as pocket gophers that excavate burrows may save energy by back-filling burrows that are no longer used rather than excavating soil to the surface (Smallwood and Morrison, 1999), which is effective in complex 3-D burrow structures but may not be an option for robots burrowing in straight tunnels, e.g., for laying cable.

**FIGURE 3:**
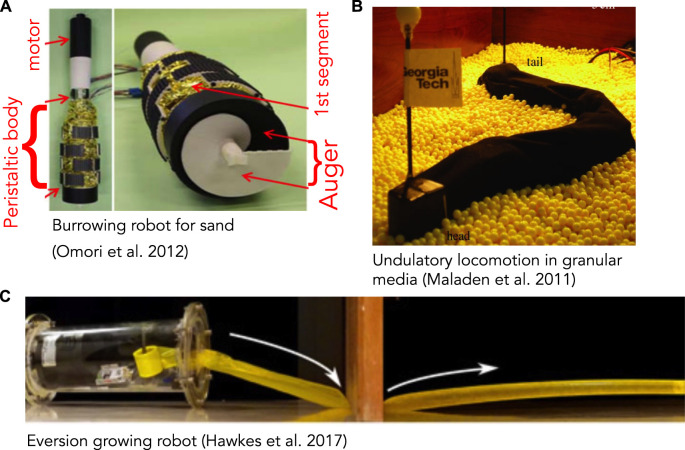
**(A)** Burrowing robot with an auger at the end for excavating and peristaltic segments (yellow) to move forward and anchor. The auger is driven by a motor and excavated soil moves up through the peristaltic segments and discharged at the narrower region above (reproduced from Figure 18 of Omori et al. (2013) with permission). **(B)** Undulatory robot based on locomotion by the sandfish lizard that swims through granular media (reproduced from Figure 4A of Maladen et al. (2011) with permission). **(C)** Soft, growing robot that navigates its environment through growth, by pressurizing a thin-walled tube to evert and extend it (reproduced from Figure 4C of Hawkes et al. (2017) with permission).

Robots that have successfully created space to burrow through solid substrates tend to be larger and demonstrate design factors and solutions that differ from biological ones. While designing a robot that can excavate and transport the substrate may seem more challenging than merely pushing it aside, the first worm-like robot to traverse sand used a rotating drill-like head to pass granular media through the body (Figure 3A) (Omori et al., 2013; Isaka et al., 2019). This design is quite different from live worms, and instead followed existing drilling technology; the robot is also larger than most animals, with 13 cm diameter. An advantage is that in addition to excavating, the head itself can help propel the robot. A more animal-like mouth, for example like (Mangan et al., 2002), would be a possible way to “ingest” material into the interior of the worm body and then transport through the burrow. However, additional design work would be required to incorporate such a head on a mobile robot, and furthermore, having to excavate material may limit the practical length of the burrow.

**FIGURE 4:**
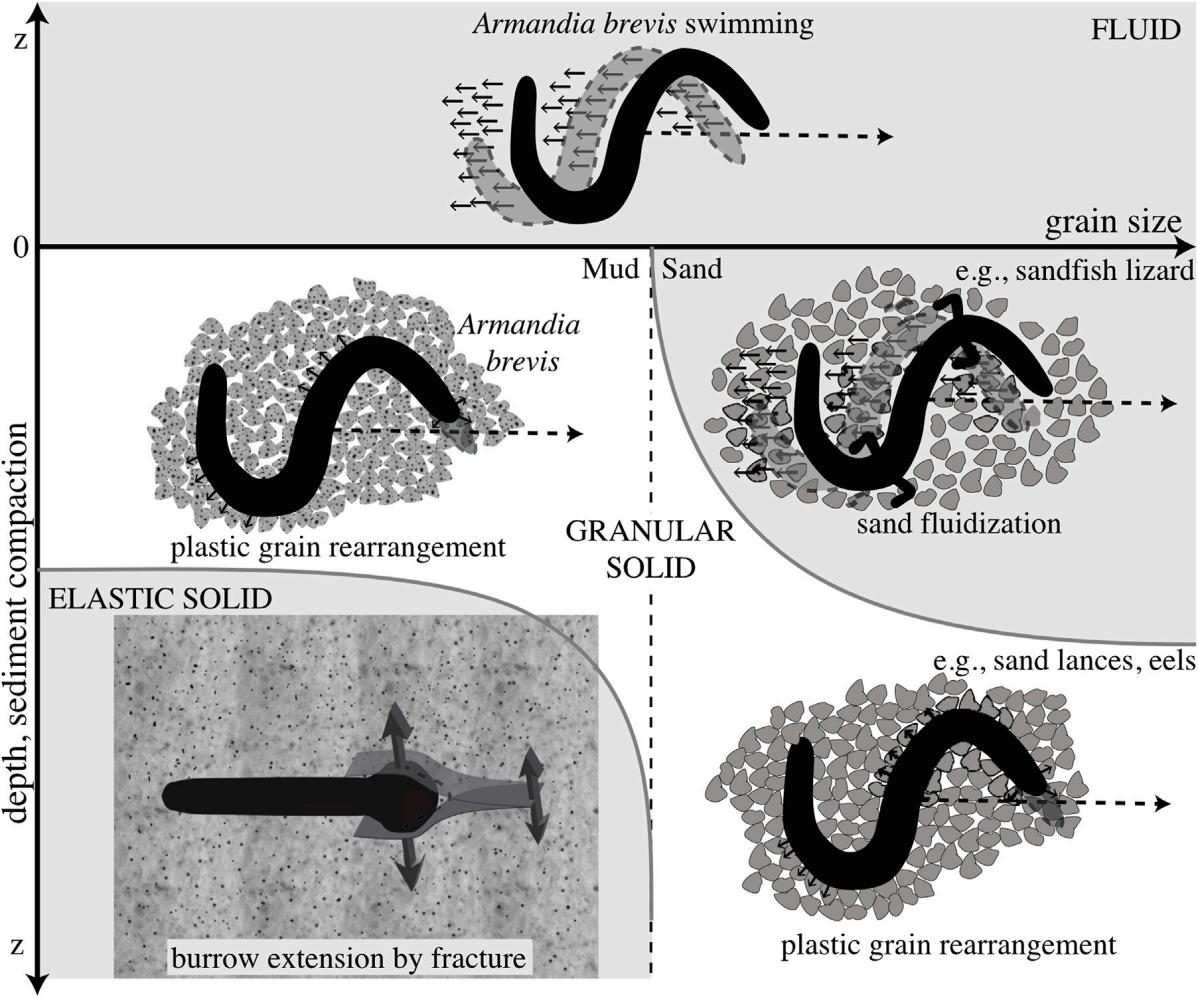
Schematic diagram of different mechanisms of burrowing in idealized muds (left) and sands (right). Dotted line indicates a later time, and the differing mechanics of the media are indicated. Swimming through a fluid is distinguished from plastic grain rearrangement of granular solids in that the body slips backwards in fluids but follows a path without slipping in solids. These mechanisms are distinguished from burrowing by fracture in elastic muds. Reproduced from Dorgan et al. (2013) with permission.

Fluidization is used by some larger animals burrowing near the surface of granular sands. The sandfish lizard swims through dry desert sands by undulating its body to apply thrust to the fluidized granular medium (Maladen et al., 2009) (Figure 4). A robot based on the sandfish successfully swims through a granular medium, although with more backward slipping than observed in animals (Figure 3B) (Maladen et al., 2011). Numerical simulations indicate that increasing the number of segments in the robot (from 7) to obtain a smoother sinusoidal curve would increase performance; backward slipping reached an asymptote at ∼15 segments. Similar undulatory behaviors are used by snakes burrowing in more consolidated sands (Sharpe et al., 2015) and small worms burrowing in surficial muds (Dorgan et al., 2013) (Figure 4). Swimming sandfish slip backwards as they apply force to the fluidized sand; this can be visualized as non-overlapping undulatory waves. In contrast, snakes and worms burrowing in muds show little or no slipping and body shapes overlap over time, indicating that the substrate is deforming like a solid rather than flowing like a fluid (Dorgan et al., 2013; Sharpe et al., 2015).

Fluidization also is used by rapidly burrowing mole crabs that live in wave-swept sandy beaches; they use rapid movements of their legs to push grains backwards as they quickly burrow down into the sand (Trueman, 1970). Mole crabs have inspired robotic solutions that excavate with legs (Russell, 2011; Teers et al., 2022). This burrowing mechanism is limited to the surface layer of sand; mole crabs generally burrow only deep enough to cover themselves but leave feeding appendages exposed. This is similar to the deepest depths achieved by a mole-crab inspired robot with counter-rotating legs in beads (Treers et al., 2022). Authors suggest that achieving deeper depths may be possible, even in natural soils, with a combination of higher torque actuators, additional fluidization strategies, or local inertial effects.

Fluidization is a feasible engineering solution to creating space in soils and sediments, either through hydraulic fracturing of cohesive muds or suspension of grains under increased fluid pressure. However, engineering challenges will include maintaining fluid flow for long or untethered systems, and controlling outputs of such flows since unintentional tunnels can be created. If autonomous locomotion is not a major concern, then tethers could allow fluidization. Alternatively, very localized flow might be achievable on small scales, e.g., through discontinuous inflation and deflation to create small, focused jets of water to facilitate hydraulic fracture.

#### 3.2.3 Intermediate-sized burrowers

**FIGURE 5:**
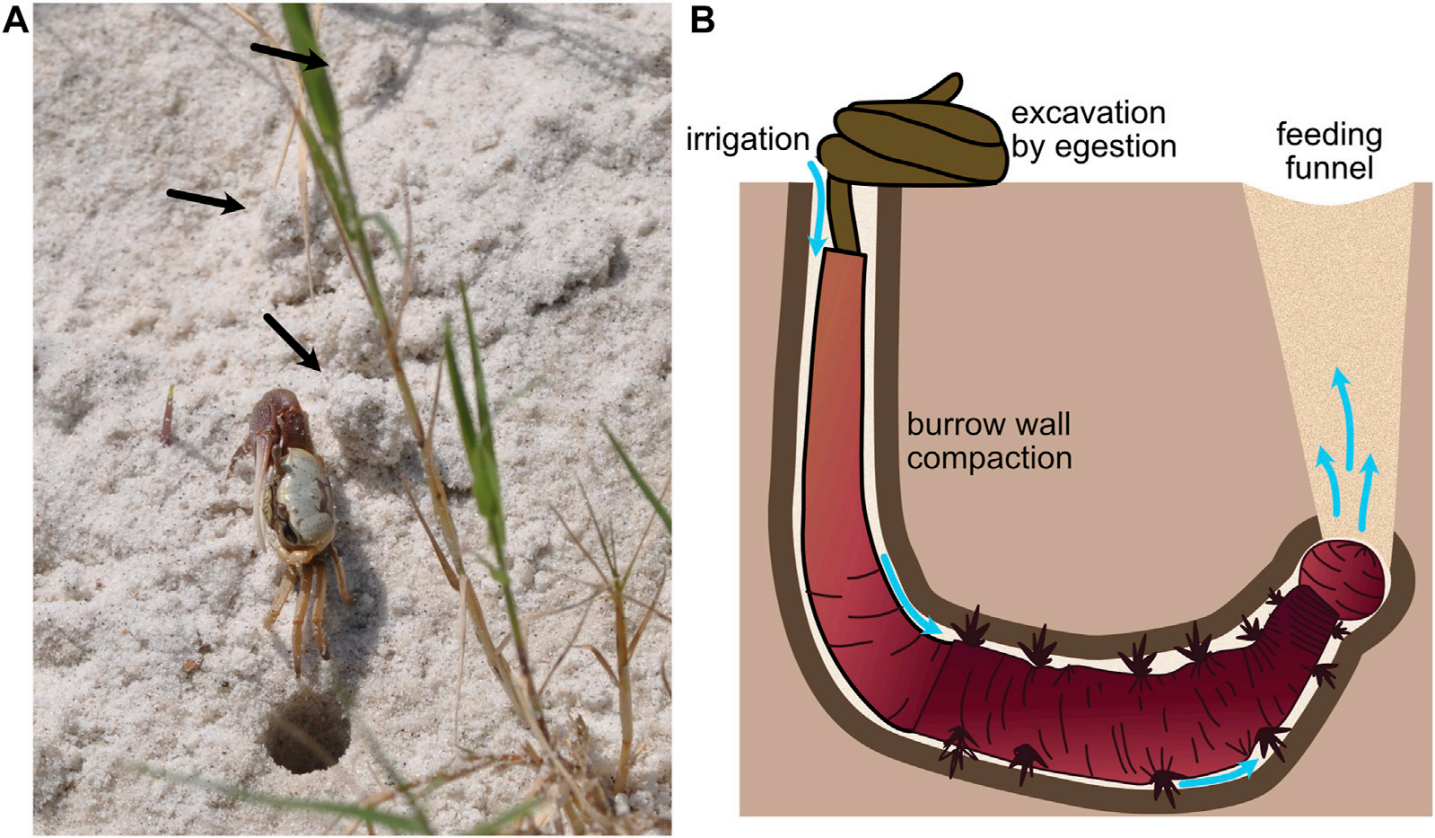
**(A)** Fiddler crab showing excavated material (black arrows) around the burrow (burrow opening ∼1 cm diam). **(B)** Schematic of arenicolid polychaete, showing burrow wall compaction and excavation by ingestion of sediments at the anterior of the burrow and egestion at the sediment surface in characteristic fecal mounds. The worm uses peristalsis to pump water from the tail shaft anteriorly through the burrow (blue arrows). Feeding causes collapse of overlying sediment to form a feeding funnel. Worm drawing by Erin Kiskaddon.

Intermediate-sized burrowers, with burrow diameters on the order of 1 cm, use both the fracture and compaction demonstrated by smaller animals and excavation of larger animals. Results from a modeling study suggest that fiddler crabs excavate sandy sediments to make burrows (2 cm diameter) (Figure 5A), but that in lower-elevation, muddier sediments, they construct burrows by compacting some sediment into burrow walls and excavating some sediment (Huang et al., 2007). This combination of compaction and excavation likely balances the energetic costs of compacting sediments, which becomes increasingly difficult as burrow diameter increases, and transporting sediments out of the burrow. Compaction also reinforces burrow walls to prevent collapse. Arenicolid polychaetes (with diameters ∼5–10 mm) live head down in J-shaped burrows that they irrigate using direct peristaltic waves to drive water down toward their heads (Figure 5B). In sandy sediments, increased hydraulic pressure fluidizes the sand around the head and water percolates up through the sand, whereas in muddier sediments, hydraulic fracture occurs, resulting in distinct plumes of water (Volkenborn et al., 2010). In both substrates, space is created for the worm to move forward. Although these worms lack rigid structures for scraping, they excavate sediment by ingestion at depth and egestion at the surface. Their peristaltic movements also compact burrow walls, although how much of the burrow volume is compacted and how much is excavated and whether this differs across sediment types have not, to our knowledge, been measured. This transition from compaction to excavation with increasing burrower size makes sense given the mechanical behaviors of soils and sediments. As burrower size increases, compaction becomes increasingly difficult, likely due to non-linear force-deformation curves of soils. As soils are compacted, they become stiffer, and more force is required to deform the soil further.

A robotic probe that has been demonstrated at intermediate size is the Mars Insight project with uses a hammering mechanism (Olaf et al., 2019). In this case, the advancing motion creates the space, a strategy that depends on a stiff rigid body.

#### 3.2.4 Scaling considerations in robot design

Peristaltic robots that span a broad range of scales have been built, but most crawl or move through tunnels and have not yet solved the problem of creating space. Many current worm-like robots are scaled for traversing pipes (Liu et al., 2022). Larger sizes with dinner-plate diameters are more convenient for manual assembly (Boxerbaum et al., 2012) and sensorization (Kandhari et al., 2018, Wang et al., in revision), but creating space in substrates for these larger robots will be difficult. Small peristaltic robots can be made at endoscope scale (Mangan et al., 2002; Adachi et al., 2011). However, at this scale, it is difficult to add sensors and additional degrees of freedom and to advance from tethered to autonomous locomotion.

To our knowledge, soft-bodied worm-like burrowing by crack propagation has not been implemented robotically, and the small sizes of animals that do it indicates that this strategy would require making smaller robots than most currently being developed. Fortunately, new fabrication approaches, including increasingly small 3D printing, origami, molding and material science, make it more feasible to manipulate and assemble small structures (e.g., Niiyama et al., 2022). Some structures incorporate actuation and sensing, e.g., though pressurized fluid or force resistive materials, which potentially further enables size reduction. However, as the complexity of fabrication and material selection increases, devices are increasingly optimized and fabricated as complete systems, it becomes all the more important to understand animal behavior as a guide. For example, peristaltic robots with diameters on the order of 1 cm would be feasible to build, and a better understanding of how much animals of that size excavate *versus* compact burrow walls would be helpful in solving the problem of creating space.

A major challenge in developing small robots is that in addition to effectively moving through the environment, many applications require robots to be autonomous. Autonomy is more challenging for smaller robots. For most applications of burrowing robots, however, being tethered does not present as much of a problem as, e.g., for swimming robots that explore much larger areas. Given the challenges of creating space to move into, it may be more practical to focus development efforts on making tethered robots smaller and more effective rather than autonomous. It is possible to make tethered robots much smaller by putting the electronics in a larger case that could be deployed on landers or AUVs. Variability in sediments is much greater vertically (with depth in sediment), than horizontally; a potential strategy for surveying sediment properties is with a swimming or crawling robot that covers horizontal distances more efficiently and then probes sediments vertically with a small tethered worm-like robot.

An additional incentive for making small, tethered robots is their potential application to biological questions. Performance of robots with different strategies can help better understand burrowing strategies. For example, at what body size (diameter) do burrowers and should burrowing robots switch from compaction to excavation? How does the performance and energetics of different burrowing strategies change across different sediment types and with depth in sediments? Burrowing mechanics and behaviors of burrowers depend strongly on the material properties of muddy sediments, specifically the fracture toughness and stiffness (Dorgan et al., 2008). Measurements of these properties in natural sediments are very limited, and how they relate to more broadly measured sediment properties such as grain size, porosity, and organic content, is poorly understood. Robots that could measure these properties would be very useful in assessing habitat differences across different types of sediments. Measurement of the forces that worms need to apply to burrow in different substrates could allow estimation of energetic costs and trade-offs in burrowing through different sediment types as well as with depth in the sediment.

### 3.3 Design of burrowing animals and robots

These transitions in the mechanisms of burrow construction as burrow diameters increase show that the challenge of creating space while burrowing depends on size, specifically the diameter, of the burrower. It is worth noting, however, that these transitions in mechanisms of burrow extension with burrower diameter are accompanied by substantial differences in the morphologies of burrowing animals. Soft-bodied burrowers are mostly small enough to extend burrows by fracture or compaction, which is consistent with their ability to expand their bodies in different ways to apply compressive stress to burrow walls. Many larger excavators use hard scraping structures that need to be articulated, e.g., with a more complex exoskeleton (crabs, insects, spiders) or an internal skeleton (mammals). Many soft-bodied burrowers are also deposit feeders and excavate by ingesting sediments and egesting them on the sediment surface (Figure 5B), although their mechanisms of freeing particles for transport are fundamentally different since they lack structures to scrape. An exception is the ice cream cone worm (Pectinaridae) that uses thick, golden spines that extend anteriorly from its head to scrape sediments (Rouse et al., 2022).

Engineered solutions have traditionally been harder and stiffer than soft animals in order to apply sufficient force to compact, fracture, or excavate soil. Rigid materials provide more strength, and so can be used to drill (Figure 3A; Omori et al., 2013), penetrate (Naclerio et al., 2021) or swim (Maladen et al., 2011) through sediments. Another option for penetrating solid soils and sediments is to use a tapered head as a wedge, as in steerable needles (Okazawa et al., 2005). However, rigid materials do not fill irregular spaces as well. Pressurizing or jamming soft structures can increase stiffness, but there are limitations in the amount of available pressure without an external vacuum or compressor and in the material strength before failure. Soft robots may be effective at extending burrows by fracture if they can be fabricated in small enough sizes, but if they need to excavate, they may need rigid structures in the anterior.

Some engineered robotic heads avoid the challenge of repeatedly advancing the head by continually growing the front of the robot. A worm-like head needs to alternate between sliding through material and expanding against it, which requires toughness and low friction. However, the softer the material, the more wear becomes a problem in engineered materials. While biological components constantly heal and grow, most engineering materials do not. Plant-inspired robots may provide alternate solutions, in which plastic is continually added to the tip (Sadeghi et al., 2017). In particular, a new class of eversion robots have similarities to the eversion shown in worms. While worms use eversion of a portion of their bodies, everting and retracting with each step, engineers have taken this a step further and have created an entire soft body that rolls out (everts) from a fixed base (Figure 3C) (Hawkes et al., 2017; Putzu et al., 2018). The advantage of this approach is each part of the body contacts only once and never has to translate relative to the contacting substrate. The result is a long tube of air between start to end points. This and other robots (Walker, 2015; Coad et al., 2019) that grow in length will likely continue to derive inspiration from vines and roots.

### 3.4 Burrowing strategies depend on depth

In marine sediments, burrowers stay close to the sediment surface, so strategies used by animals to create space in soils and sediments may become less effective at depths >10–15 cm. There are several reasons for animals to stay near the sediment surface. First, animals need oxygen, which is rapidly consumed by the microbial community in sediments, resulting in a lack of oxygen below a few millimeters (Middelburg and Levin, 2009). Infaunal worms deal with this problem by irrigating their burrows, bringing oxygenated water from above down into the burrow or by extending external gills up out of burrows. Additionally, many burrowing animals feed on sediments and digest the organic material coating mineral grains (Jumars et al., 2015). The quality of organic matter is often highest at the sediment surface, where fresh phytodetritus is deposited from overlying water. Burrowing, ingestion, and egestion mix sediments, a process called bioturbation (Meysman et al., 2006). The mixed layer of sediments is on average about 10 cm deep (Boudreau, 1998). Although burrowing happens at greater depths, the majority of burrowers stay in this upper layer of sediments. There are indications that even within this upper 10 cm layer of sediment that the mechanics differ between a surficial, unconsolidated layer and a deeper, more compacted layer (Thomsen and Gust, 2000). In granular materials, fluidization primarily occurs in the upper layer. Tip-based airflow reduced resistive forces for a robot burrowing in dry sand, but this effect was limited to a critical depth of 8–10 cm, below which forces increased sharply (Naclerio et al., 2021). In elastic muds, the surface layer (∼1 cm) often comprises looser aggregates that burrowers can push aside (i.e., by plastic deformation or compaction), whereas burrowers fracture more consolidated subsurface muds (Figure 4). For larger excavators, deeper, more consolidated sediments likely require greater forces to compact burrow walls, and the distance to transport excavated material increases. Thus, burrowing is likely more costly, but how depth affects the efficiency of compaction *versus* burrowing has not been studied.

Penetrating the sediment is an important consideration for animals or robots that crawl or swim as well as burrowing. Considerable research has focused on how animal morphologies and substrate type affect the speed and efficiency of burrowing into sediments. Burrowing quickly is particularly important in sandy beaches where waves can transport animals that are unable to burrow within a wave period. Both the clam, *Donax*, and the whelk, *Bullia*, burrow in the swash zone of beaches; both can burrow only in fully saturated sand and use water jets to fluidize the surface layer (Brown and Trueman, 1991). *Donax* also probes the sand with its foot when burrowing, whereas *Bullia* does not, likely because *Bullia* burrows at a shallow angle of 10°–15° at which penetrometer measurements indicate resistance is ∼10% of the resistance of burrowing straight down (Brown and Trueman, 1991). Bivalve species with more circular shells tend to rock within the plane of the shell opening and sometimes move horizontally as they try to penetrate the sediment, whereas more elongate shells such as razor clams exhibit very little rocking and burrow down more smoothly (Stanley, 1970). Penetration resistance increases with depth in sand, and the polychaete *Thoracophelia* exhibits behaviors that can be interpreted as dealing with varying penetration resistance in beach sands; worms burrow with a direct peristaltic wave and expand their head region to apply forces to the burrow walls, but they occasionally expand the entire anterior region of the body radially, presumably to apply larger forces along a longer region of the body (Dorgan, 2018). Unfortunately the penetration resistance in experimental tanks was not well standardized, so whether these expansions are in direct response to increased penetration resistance could not be directly tested.

## 4 Burrowing challenge 2: Moving forward with a soft body

Once new space is made in the substrate, the burrower needs to move its body forward into the new section of burrow. This problem of moving forward in confined spaces can be broken down into two distinct considerations in development of burrowing robots: first, body movements to elongate segments forward (e.g. by contracting circular muscles) and, second, strategies to anchor against the burrow walls. Anchoring, which can be achieved by expanding radially or by other engagement mechanisms, is discussed in the next section. Moving forward and anchoring are tightly coupled, with a single segment cycling through anchoring and advancing phases. Similar to walking, in which the leg motions determine the ground reaction forces, the coordination of moving forward will determine the anchoring forces for the worm. However, we separate them because while anchoring is needed for moving forward, the magnitude of the anchoring forces required should be largely dominated by the problem of creating space.

The basic challenge of moving forward with a soft body is in controlling the deformation of the body. For a typical wheeled or articulated rigid robot, the way the center of mass moves relative to the footprint is well defined by kinematics. For example, the wheel rotates, and the center of mass of the body moves forward. The components are discrete with relative motion and are limited to rotation about one axis. In worms, however, each moving segment needs to advance *via* deformation that is often more distributed throughout the body, e.g., with a hydrostatic skeleton that has many degrees of freedom. The body has to be soft enough to undergo significant deformation, yet rigid enough to transmit forces.

### 4.1 Types of forward motion

There are several strategies to move forward through soils and sediments. Plant roots grow only at the tip, while the main part stays in place, a strategy that has recently been applied to robotics (Mazzolai et al., 2014; Hawkes et al., 2017). Burrowers with limbs and either internal skeletons or rigid exoskeletons, e.g., mammals and crabs, use appendages rather than body expansions for locomotion. Thus, movement into newly created burrow space is similar to crawling or walking, although the radial walls of the burrow allow greater thrust than a flat surface. Soft animals however, will typically have fewer moving parts than required by legs and will use less material than required for growing roots or vines.

**FIGURE 6:**
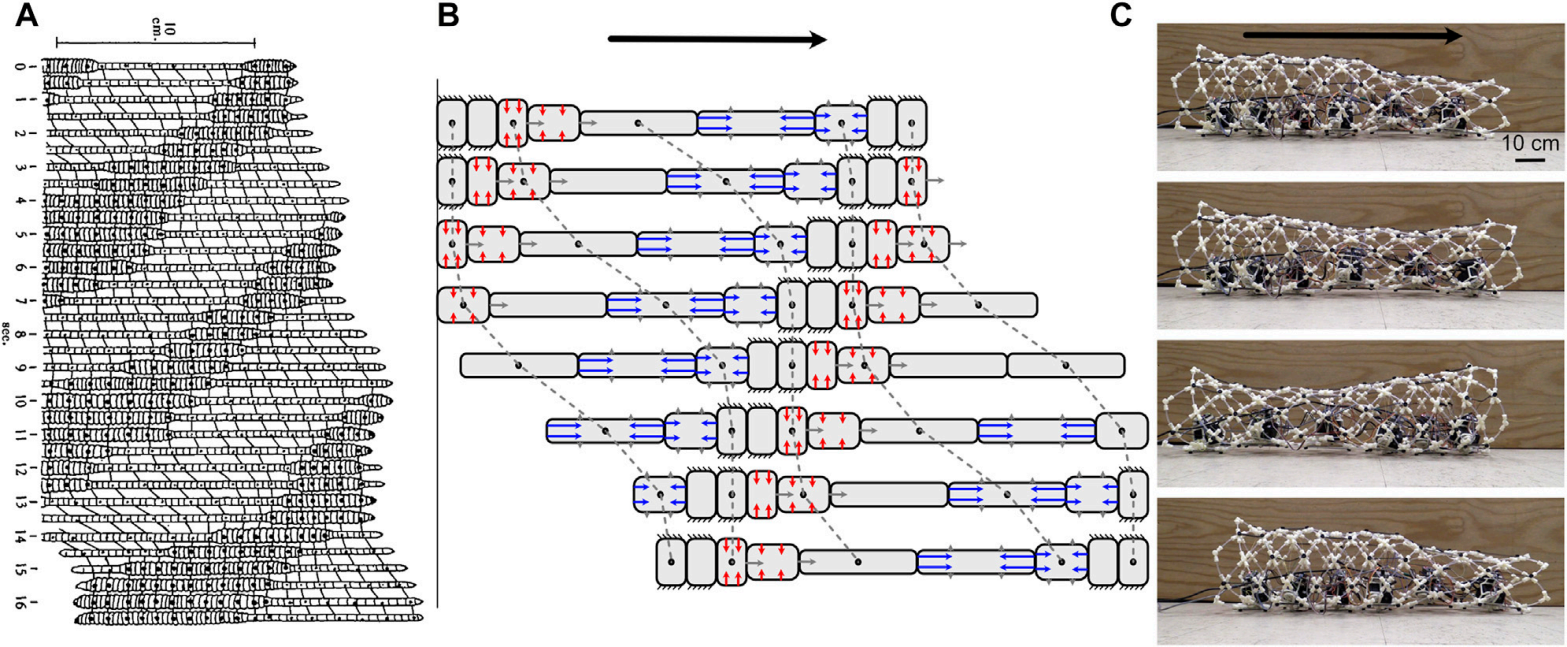
**(A)** Drawing of peristaltic movement by an earthworm showing narrow segments moving and dilated segments anchoring. Reproduced from Gray and Lissman (1938). **(B)** Diagram of simplified peristalsis mimicking an earthworm but with fewer segments. Anchored segments are indicated by black lines extending; also shown are segments in which circular muscles (red) are contracting to elongate the segment (against a posterior anchor), and those in which longitudinal muscles (blue) are contracting to shorten the segment (against an anterior anchor). In both diagrams, peristalsis is retrograde (the wave of contraction travels posteriorly), and segments have a constant volume (assuming a cylindrical shape in B). Dots connected with dashed lines show the same segment in multiple frames. **(C)** These peristaltic motions can be duplicated in robots, as in CMMWorm (images by K. Daltorio).

Live worms and some robot worms are filled with a pressurized internal fluid. In live worms, the fluid is typically self-contained to form a hydrostatic skeleton. The pressure is matched to that of surrounding water, enabling them to operate in environments that would require thick pressure vessels or saturation diving for humans. In hydrostatic skeletons, force is transmitted through muscular contraction against the fluid-filled and therefore incompressible body cavity (Kier, 2012). Muscle contraction thus changes the shape of the hydrostat, e.g., contraction of circular muscles makes a cylindrical hydrostat longer and thinner, and contraction of longitudinal muscles makes it shorter and fatter (Figure 6). Inextensible fibers and stretchy connective tissue can limit the changes in body shape and increase internal pressure; for example many cylindrical hydrostats have helical fibers that limit radial or axial elongation, depending on the angle of the fibers (Koehl and Quillin, 2000).

#### 4.1.1 Dual anchor locomotion

Animals and plant roots use several strategies to move forward through soils and sediments. The simplest approximation of movement through a burrow by soft-bodied animals is the dual-anchor model, in which a penetration anchor holds the body in place while the anterior end moves forward, then the anterior end expands to create a terminal anchor that holds the body in place while the posterior is pulled forward (Clark, 1964). The dual-anchor system is exemplified by clams, which alternate expanding their muscular feet to create a terminal anchor and their shells to create a penetration anchor (Trueman, 1968) (Figure 1B). This mechanism is also used by burrowing snails (Trueman and Brown, 1992) and many diverse worm-shaped animals that expand their anteriors through eversion of mouthparts or through contraction of body wall musculature (Dorgan et al., 2006).

**FIGURE 7:**
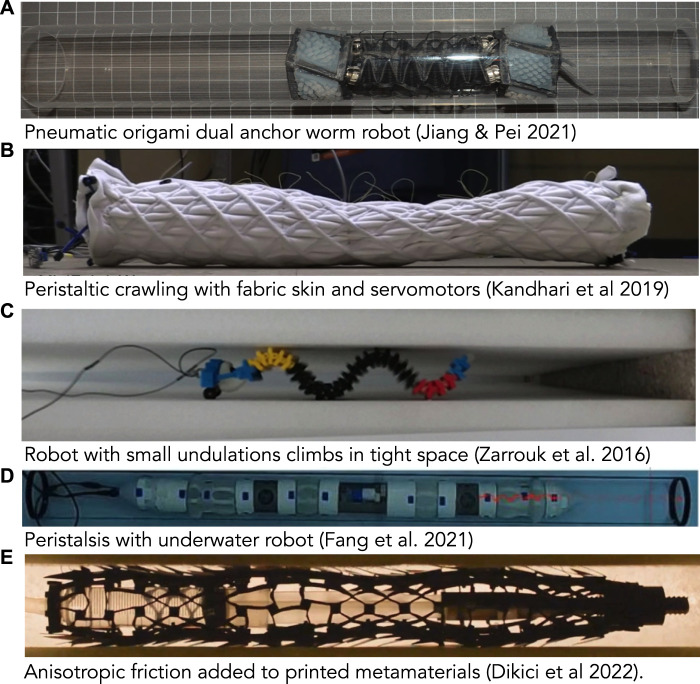
Photos of worm-inspired robots. **(A)** Pneumatic origami, dual-anchor robot (reproduced from Figure 4 of Jiang and Pei (2021) with permission). **(B)** Peristaltic crawling robot comprising fabric skin over servomotors (Kandhari et al., 2019b). **(C)** Undulating, worm-inspired robot crawling up between vertical walls with small undulations acting as anchors (reproduced from Zarrouk et al. (2016) with permission). This image was rotated to align with other images in the figure; the robot is moving vertically (toward the left) **(D)** Peristaltic robot with paired expanding and elongating segments moving through a tube underwater (toward the right) (Fang et al., 2021). **(E)** Peristaltic robot made from printed metamaterials with chaetae-like extensions added to create anisotropic friction (Dikici et al., 2022).

An advantage of dual-anchor locomotion in robotic design is that elongation can be achieved *via* a wide array of strategies because the elongating part does not need to alternatively anchor. Thus, the recipe for a dual-anchor robot is an elongating segment sandwiched between two anchoring segments (Figure 7A; Jiang and Pei, 2021). All three segments can be pneumatic (Liu et al., 2019; Wang et al., 2021; Connolly et al., 2015), or elongation and anchoring can be achieved completely differently, e.g. by suction and pneumatic means (Zhang et al., 2021). To create a longer robot, pairs of elongating and anchoring segments can be chained together (Zarrouk et al., 2012). A disadvantage of this type of locomotion is that only certain parts of the body are designed to contact the substrate, while others do not. In particular, if the burrowing medium tends to collapse back onto the worm, the elongating segments should be close in diameter to the anchoring segments. However, if tunnel surfaces are uneven, larger-diameter moving segments experience greater frictional forces that can interfere with the steps.

#### 4.1.2 Peristaltic locomotion

A more complex anchor system is peristalsis, in which waves of contraction or expansion travel down the body creating multiple anchors that travel with the waves. Both peristaltic and dual-anchor locomotion are intermittent—part of the body moves while another part is stationary, resulting in incremental forward movement. Peristalsis achieves this motion with a smooth continuous wave, however, in which every segment participates in moving forward, and every part of the exterior can anchor.

The classic example of peristaltic locomotion is of earthworms, in which circular and longitudinal muscles in the body wall contract antagonistically (Gray and Lissmann, 1938; Elder, 1980) (Figure 6). When the circular muscles of a segment with constant volume contract, that segment gets longer. Each segment goes through a cycle of anchoring, elongation, and contraction (Figure 6). If there is no slip at anchor points, then the segment moves forward during both elongation and contraction (Figure 6B).

Robots can locomote peristaltically as well (Figure 7). They can use antagonistic actuation (e.g. using radial and zig-zagging longitudinal tendons) (Horchler et al., 2015). Alternatively, they can use circular actuators alone (which are simpler) and use passive springs (Kandhari et al., 2018) or even knit fabric skin (Figure 7B; Kandhari et al., 2019a) to provide the forces to return to the elongated state. Peristaltic locomotion works on flat ground, but is even more valuable in a confined tube (Figure 7D).

Some worms, including annelids like earthworms, are segmented, with separate volumes of fluid in each segment. Muscular walls called septa prevent flow of internal fluid between segments. An advantage of septa in burrowing is that they allow pressure to vary along the length of the animal, so that only segments involved in locomotion need higher pressure to allow the muscles to act antagonistically. In addition, if the segments are separated by septa, damage to part of the animal is localized. For example, if a fish bites the tail off a worm with septa, the hydrostatic skeleton at the head end still functions, and the front part of the worm can escape. A limitation is that when segment volume is fixed, the worm has less flexibility in movements that will result in forward progression. Forward locomotion is only possible when waves of contraction travel posteriorly along the worm’s body; this is called retrograde peristalsis (Elder, 1980) (Figure 6).

**FIGURE 8:**
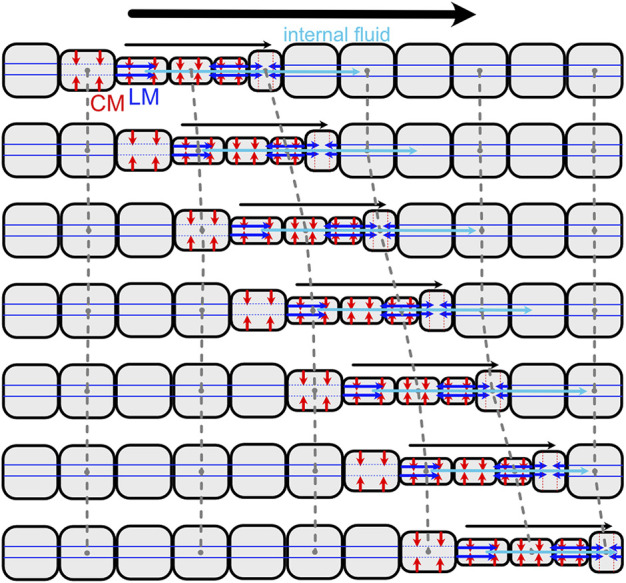
Diagram of direct peristalsis, following peristalsis by *Thoracophelia mucronata* (Figure 2). The wave of circular muscle contraction travels toward the head, starting with contraction of the circular muscles (CM; red) to reduce the diameter, then contraction of the longitudinal muscles (LM; blue) to pull the narrow segment forward. Internal fluid (cyan) moves between segments, e.g., being pushed forward as the contractile wave moves forward.

Loss of septa in part of or all of the body has occurred several times in annelids, and allows greater flexibility than classic retrograde peristalsis used by earthworms. Forward movement can also be achieved using direct peristalsis, in which the waves travel in the forward or anterior direction (Elder, 1980). For example, a wave of contraction (thin segments) travelling forward pushes body fluid forward, resulting in forward head progression (Figures 2, 8). Direct peristalsis is also used for burrow irrigation: the worm *Arenicola* lives head down in burrows and uses a wave of expansion traveling anteriorly like a piston to drive flow forward in the burrow (Wells, 1966). Irrigation by the annelid, *Sabella*, that lives in a tube with its head up and tail down, is similarly achieved using expanded segments to drive water like pistons, but in this case the peristaltic wave is retrograde and the body is separated by septa (Mettam, 1969). Movement of fluid between segments with incomplete septa also allows for greater flexibility in the relative lengths of the anchoring and moving segments. Some worms that live in very soft muddy sediments have mostly open body cavities; a thin band of contracted segments travels anteriorly, moving internal fluid forward to apply a relatively low pressure along a relatively large region of the body wall, resulting in enough force to extend the burrow by fracture (Hunter et al., 1983; Dorgan et al., 2016). Direct peristalsis is also used by worms burrowing in granular beach sands that require larger forces to compact the surrounding sand: the peristaltic wave pushes internal fluid forward to increase pressure in the anterior of the worm (Figure 2) (Dorgan, 2018).

Fluidic worm robots are typically actuated by varying the volume of fluid in a small number of chambers. Without additional muscles, the volume and pressure are coupled. Some soft robots regulate many segments by setting precise volumes of compression cylinders (Marchese et al., 2016), others regulate pressure with a compressor which can be small enough to be on board (Tolley et al., 2014). Usually, the fluid is air, but underwater the natural choice would be water, as in Ishida et al. (2019). In other cases, the structure of the body is designed to mimic a hydrostatic constraint (Vaidyanathan et al., 2000; Chatterjee et al., 2017). These robots have fluid-filled segments with SMA (Shape Memory Alloy) muscles that provide actuation. To better mimic the adaptability of behavior in burrowing worms, fluidic control may need to be combined with other types of artificial muscles.

#### 4.1.3 Undulation for locomotion

**FIGURE 9:**
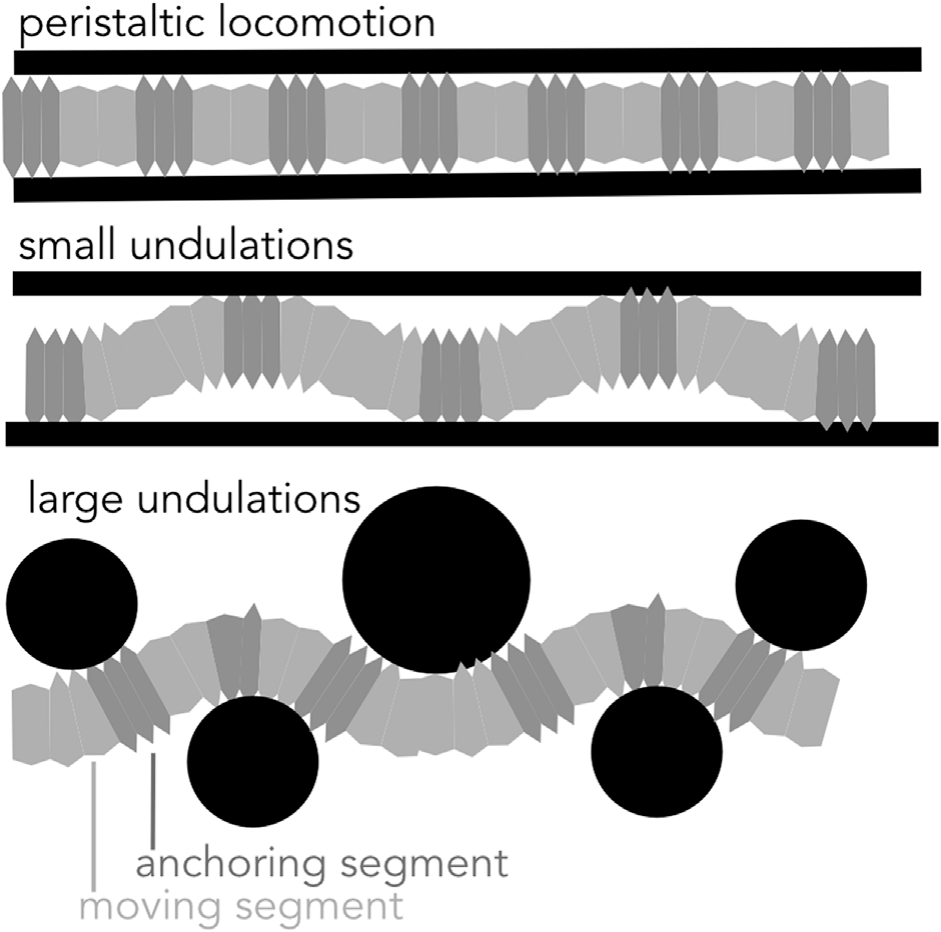
Diagram of peristaltic and undulatory movements. Soft-bodied burrows create anchors to generate thrust in a cylindrical burrow using peristaltic expansions and small undulations. Larger undulations result in a meandering path and are effective in moving around obstacles.

Another way of moving forward is through bending the body, either with small undulations within a straight burrow or with larger-amplitude undulations that create a meandering burrow (Figure 9).

If the burrow is larger in diameter than the largest segment diameter, small oscillations in the body result in a wave of “anchors” that apply forces very similar to those in peristalsis (Dorgan et al., 2007). For example, nereids (Figure 1A) use longitudinal and oblique muscles to achieve small-amplitude undulations (Figure 9). Instead of radial expansion of the segments, the body bends laterally to brace on the burrow wall (Dorgan et al., 2007). This strategy likely reflects the more general locomotory capabilities of these worms; nereids use undulations to swim, crawl through complex substrates like oyster reefs, and irrigate burrows (Gray, 1939). A disadvantage of small undulations compared to peristalsis is that peristalsis more effectively reduces friction for moving segments, which are narrower than anchored segments.

Robotically, small undulations would have advantages, mainly in increasing the range of burrow diameters that a single robot could traverse. A worm robot that undulates using actuators can successfully climb up vertical pipes (Figure 7C; Zarrouk et al., 2016). For example, a large burrowing head could be followed by a narrower body. Furthermore, some soft actuators are inherently better at bending, e.g. bilayers, see review at (El-Atab et al., 2020), and some soft robots are already adapted for swimming (Baines et al., 2021), and thus multimodal locomotion is plausible. A challenge is in getting the coordination correct so that the segments do not jam in adapting to differing burrow sizes.

Another strategy of moving forward is through large undulations of the body (Figures 3B, 4, 9), in which forward motion occurs simultaneously with creation of space, and the entire body moves at once (Maladen et al., 2009). This mechanism is limited to environments in which creating space is relatively easy, i.e., surface unconsolidated muds or granular sands (Maladen et al., 2009; Dorgan et al., 2013), or by larger animals such as eels that are capable of applying larger forces (Herrel et al., 2011). Friction between the body wall and the sediment is greater than for animals that use the dual-anchor system, peristalsis, or peristaltic-like undulations that reduce friction by moving narrower body parts while thicker body parts anchor. Undulations have been successfully applied in robots. Sandfish-like robots move in loose granular media (Figure 3B; Maladen et al., 2011). Robotic snakes can sidewind on sand (Marvi et al., 2014).

### 4.2 Geometry of peristaltic burrowers

Elongate bodies allow burrowers to create a small-diameter burrow through substrates, so it is unsurprising that many burrowers have long bodies—but how long is needed for effective peristaltic movement? A starting point for length requirements can be found in the design of crawling worm-like robots (Kandhari et al., 2021). At least three segments are needed for forward movement. With only three segments, however, the robot will tip under gravity if unsupported on all sides by an inner burrow wall. Many robots have four segments, enabling a pair of segments to extend and retract between two anchoring segments. As the number of segments increases, the number of moving segments can increase, thus increasing the overall speed of the robot. For example with six controlled segments, a continuum wave can be established in which 80% of the body is moving at once. However, gravity limits the number of segments that can be supported above the ground. While larger body deformations can lift the segments off the ground, there is an energetic cost of this deformation. Thus, for energetic efficiency, more segments should be kept on the ground, with efficiency leveling off at around 10 segments total (Kandhari et al., 2021).

Most burrowing annelids have considerably more than 10 segments, but their lengths likely reflect constraints other than locomotion, e.g., fitting all the internal structures needed into an elongate body. Several worms exhibit transitions in body wall musculature between an anterior region in which peristalsis occurs and a posterior, less muscular region that suggest that the anterior 9–10 segments are used in peristalsis (Law et al., 2014; Grill and Dorgan, 2015) (Figure 2). Some deposit-feeding cirratulid worms have muscular anterior and posterior ends with a less muscular middle region and can burrow both forward and backward (Che and Dorgan, 2010b). Many worms are deposit feeders that ingest low-quality sediment and have complex digestive strategies to remove organic matter from minerals and break down and assimilate the food (Penry and Jumars, 1990). Therefore, long bodies are needed to hold guts long enough to digest food and retain digested material and gut enzymes and absorb the resulting products (Penry and Jumars, 1990).

Live worms tend to have more shorter segments than robotic worms, which has several advantages. The ratio of the length to diameter (L/D) of segments in a hydrostatic skeleton affects how muscular force production translates to forces applied to the environment (Kurth and Kier, 2014). With short lengths, longitudinal muscles need to contract only a short distance to expand the segment radially (Quillin, 1998). This enables rapid anchoring, e.g., if a predator tries to pull a worm out of its burrow. In addition, shorter segments provide finer spatial resolution, which means that the body can more precisely adapt to uneven burrows and perhaps expend less energy in anchoring. Earthworms that burrow have relatively larger longitudinal muscles and can exert larger radial forces than crawling surface-dwellers, but crawlers have larger circular muscles (Kurth and Kier, 2015), which can provide faster forward progress. Similar trade-offs will likely be involved for worm robots.

Underwater, gravity may not be a limiting consideration, so it may be possible to modify the number of segments or how many of those segments are moving simultaneously to achieve faster movements. Developing new models to account for soil elasticity and strength may help optimize peristaltic movements for speed or efficiency. Non-etheless, creating hundreds of segments as in earthworms (e.g., Kurth and Kier, 2014) does not seem required.

### 4.3 Scaling of peristalsis

Peristaltic locomotion is effective across several orders of magnitude of body size, and the small changes in the scaling of segments, movements, and forces applied as earthworms grow are likely driven by the challenge of applying forces to create space rather than for effective locomotion within a confined space. As earthworms grow, they get slightly longer and thinner, giving them a distance advantage when contracting circular muscles that results in a longer stride length (Quillin, 1999; Kurth and Kier, 2014). Quillin (2000) measured forces exerted by earthworms across several orders of magnitude in body size and found that although larger worms exert larger radial forces when burrowing than smaller worms, the difference is less than the increase following a 2/3 power law predicted from geometric similarity (Quillin, 2000). Kurth and Kier (2014) found, however, that although the cross-sectional area of the longitudinal muscle increases less than predicted from isometry, the longer segments have greater mechanical advantage, resulting in comparable force production across body sizes. Smaller worms need to exert relatively larger forces to extend their burrow by fracture (Che and Dorgan, 2010a), but larger worms need to compact burrow walls a greater distance, and most soils exhibit strain hardening, as resistance to compaction increases with distance (Kurth and Kier, 2014). These scaling differences provide insight into the functioning of peristaltic locomotion, but it is important to note that the general pattern of peristalsis is effective across orders of magnitude in body size.

### 4.4 Peristaltic robots

Building robots has helped develop understanding of peristalsis. On flat surfaces, slip is a limiting factor in achieving the desired locomotion. It might seem that increasing the coefficient of friction is the solution. In fact biology-based theories claimed that the friction coefficient limited the number of segments it would be possible to move at once (Alexander, 2003). Increasing friction coefficient, however, usually makes locomotion worse (Horchler et al., 2015). When the contact is too soft (e.g. testing on soft rubber or carpet), not only is friction high but the contact between robot and ground does not start and stop cleanly. Many examples of worm robot locomotion, however, are on smooth surfaces such as tile. Often the problem is in coordination: if the sum of segment lengths between anchors increases, either slip or unplanned deformation is more likely. On smooth surfaces, a small amount of slip occurs but does not hinder overall locomotion. Thus, if the first waveforms are ineffective in generating motion, changing the gait of the robot is typically more helpful than covering the surface with higher-friction coatings, unless anisotropic friction is introduced.

Thus, for moving forward, coordination appears to be more important than the magnitude of the anchoring forces. The key is to have some segments increasing in length at the same rate as others are decreasing. The actuation method determines the precision of this coordination. While servos can be precisely positioned, the soft structures they actuate can be highly variable. Models can be developed even for continuum arms, e.g. Marchese et al. (2016), but contacts are challenging to model accurately, limiting model-based approaches. Thermally controlled segments may be particularly difficult to coordinate, but their softness may reduce the need for precision.

Simplicity of design will also be important—designing to restrict the number and variety of actuation while still achieving the desired motion will reduce the challenges associated with design, scaling, and control. Biological worms have complex musculature, with multiple antagonistic muscles used to stabilize body movements, but simpler, robotic worms can rely on passive stiffness for return forces. If the robot is small and creating space by applying radial forces, then the longitudinal muscles may be more critical for actuation. If the robot is larger and has another mechanism to excavate, e.g., an anterior auger, then having actuated circular muscles may be more appropriate.

Even as more worm-like robots are being developed for difficult burrowing applications, inspiration from biology will be important. Live worms will be the state of the art in natural environments and can inspire new approaches to learning, scalability, and design.

## 5 Burrowing challenge 3: Anchoring

The next problem in locomotion within confined spaces is the need to anchor part of the body, which animals do for several reasons. First, anchoring is important as a step within a peristaltic or cyclic forward movement, enabling the animal to selectively generate thrust or reduce friction where needed. Second, anchoring is a key part of burrowing by fracture in muds. The evidence is that radial forces are larger at the anterior (where the head initiates the crack) than the posterior of the body during peristalsis (Dorgan et al., 2007). Large radial forces both secure a portion of the body in place and separate layers to create space. When the penetration forces required are high, and the body is soft, it is important that the anchor be as close to the front as possible. Finally, strategies to improve anchoring abilities by burrowing animals may be driven as much by predation risk as by locomotory function. Burrowing is a strategy that evolved at least in part as predator avoidance. Many burrowers experience high risk of predation, and strong anchoring can prevent the whole body from being eaten. Although quantitative data on predation rates is challenging to obtain and therefore unsurprisingly sparse, it is not uncommon to find worms that are regenerating tails or even heads (Lindsay et al., 2007) or brittle stars regenerating arms, which is clear evidence of predation.

Not all expansions are anchors nor all anchors expansions. The segments that are moving in peristalsis are usually thinner than the anchoring segments, which minimizes friction, although worms can move thicker segments to irrigate burrows, using their expanded regions like pistons (Mettam, 1969). This requires the worm to have a way to grip with the narrower part of the body; the annelid *Sabella* dorsoventrally flattens the thin segments to push the extended parapodia against the wall of its tube (Mettam, 1969).

### 5.1 Anchoring in confined spaces

Moving through a cylindrical or crack-shaped burrow differs from crawling in that animals have greater surface area against which to apply forces to anchor and generate thrust and thus rely less on gravitational forces to generate friction and thrust than crawling animals do (Dorgan, 2010). In the case of peristalsis in a confined space, forward movement is balanced by the frictional force between the anchored segment and the burrow wall. This frictional force can be increased by increasing either the normal force, i.e., pushing harder against the burrow wall, or the friction coefficient. Normal forces depend on the internal pressure of a soft-bodied animal or robot, which is tightly coupled to the stiffness of the surrounding sediment; stiffer sediments allow larger pressures assuming that the diameter of the animal or robot is fixed. The friction coefficient depends on surface properties.

The question of how soft a soft robot should be affects anchoring as well as actuation in peristaltic movements. Burrowing soft robots need to be soft enough to conform to burrow walls for traction but have sufficient pressure to deform the burrow as needed. Typically, moving through a confined space will require anchoring phases (in which the body is soft enough to conform to the surrounding burrow space, and thus achieve a traction distributed over as much of the circumference as possible) and advancing phases (in which stiffness is needed to keep the moving segments off the ground) (Kandhari et al., 2018). To make space (previous section) the head must physically move the substrate. In anchoring, however, segments are not moving the substrate necessarily but using it for traction. Displacing the segment elastically can have negative effects on progress (Zarrouk et al., 2012) by increasing slipping or can contribute to burrow collapse and thus to higher locomotion costs. Enough displacement is needed, however, to make space for the burrower. This challenge is exacerbated by variability in stiffness of the substrate; anchoring strategies that work well in stiff, compacted sediments may fail in soft muds that yield easily. An advantage of being soft bodied in anchoring in sediments that vary in their resistive strength is that the size, shape, and pressure applied by the anchor can be modified. Worms that burrow in soft muds use lower pressure applied over a longer body expansion to anchor themselves; this is achieved with a direct peristaltic wave in which much of the body anchors while a short contracted region travels anteriorly (Elder, 1973). Worms likely also rely on feedbacks from burrow wall resistance and adjust their pressure dynamically. Mechanisms to increase the asymmetric friction may help reduce the need for sensory feedbacks for robots: if the friction coefficient can be increased, then the robot can use lower pressure (resulting in a smaller normal force) that is less likely to result in burrow wall expansion.

Having a curved path also enables anchoring thrust for space-making penetration to be supported from normal forces and not only shear, as has been observed in snakes (Transeth et al., 2008). The bends observed in the bodies of burrowing animals could be occurring where there is excessive slip or where typical interior stresses are not achieved during anchoring.

### 5.2 Surface features for anchoring

**FIGURE 10:**
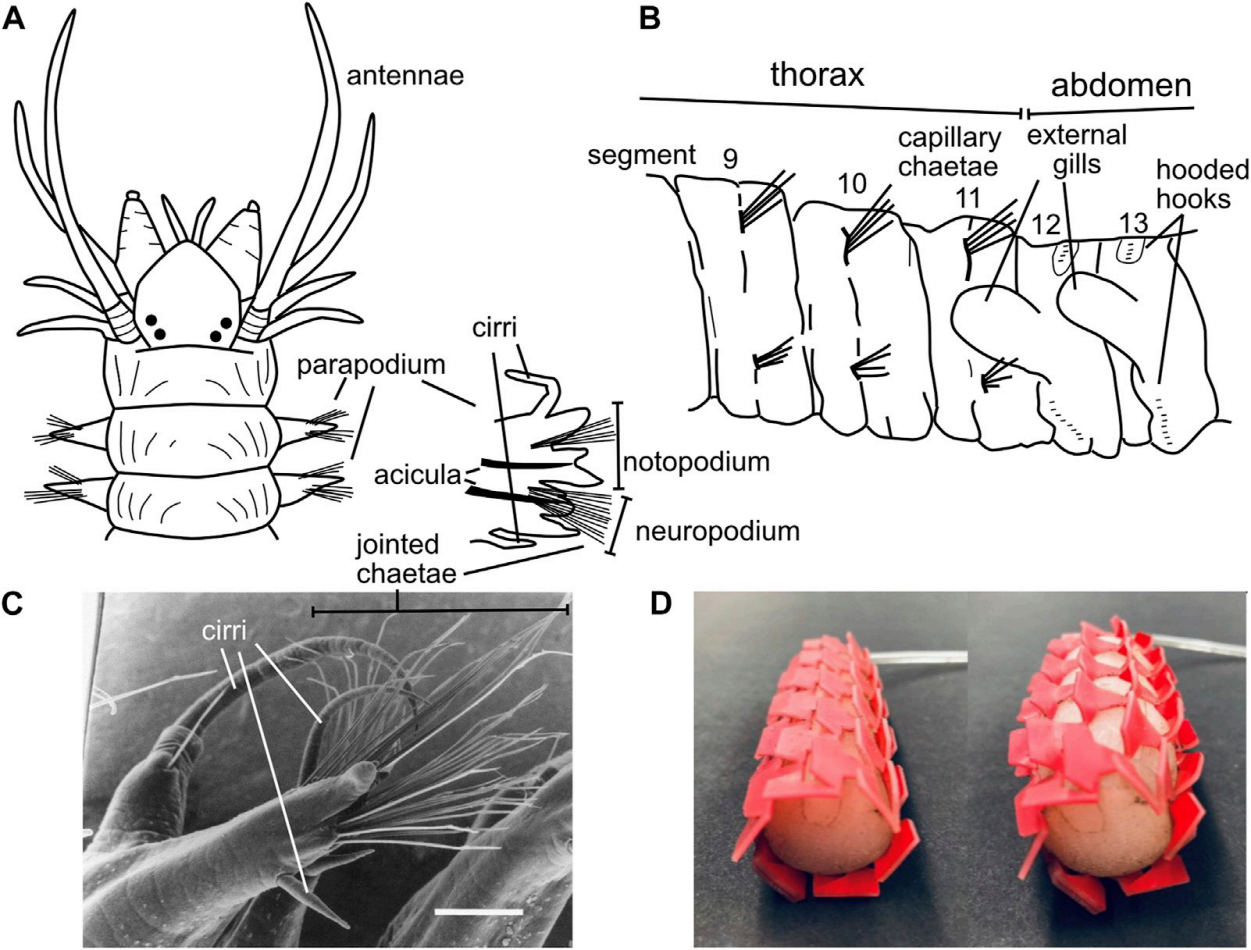
**(A)** Drawing showing parapodia with chaetae at the anterior of *Alitta succinea* (Nereididae), with one anterior parapodium enlarged to show the upper notopodium and lower neuropodium, the rigid acicula to support the parapodium, bundles of chaetae, and sensory cirri **(B)** Drawing of chaetae of *Notomastus lineatus* (Capitellidae) at the transition from the straight capillary chaetae of the more anterior thorax (anterior 11 segments) to very short, hooded hooks in the abdomen. Parapodia are so reduced as to be invisible in this drawing. **(C)** Image of a mid-body parapodium of *Ophiodromus pugettensis* (Hesionidae) from a ventral anterior view showing jointed chaetae bending back toward the posterior of the worm; scale bar is 200 μm **(D)** Image of kirigami skin on an earthworm-inspired robot showing smoother profile under low pressure (left) and ridges that pop out under high pressure to increase friction (right) **(A,B)** Drawings modified from Uebelacker et al., 1984; **(C)** reproduced from Figure 1 of Merz, 1998 with permission. **(D)** Reproduced from Figure 6 of Liu et al. (2019) with permission.

Polychaete worms, the most abundant burrowers in most muddy marine sediment habitats, are named for their “many hairs” or chaetae that extend laterally from parapodia (Latin for “foot-like”) on each segment. Bundles of hairs extend from the upper and lower parts of the parapodia, which in burrowers allows them to contact the upper and lower burrow walls (Figures 10A–C). These hairs vary considerably among species and body regions (Purschke et al., 2014; Rouse et al., 2022). While some chaetae are simple, straight hairs (termed “capillary chaetae”; Figure 10B), many have joints oriented so the tips of the hairs easily bend backwards (during forward locomotion) but not forward (to prevent backward slipping) (Merz and Edwards, 1998) (Figure 10C). When crawling, body weight is supported on bundles of chaetae, and the joints allow hairs to splay outward, increasing surface contact and stability and possibly preventing bucking (Merz and Edwards, 1998). Bundles of chaetae can be extended and retracted, allowing asymmetrical friction both directly with the chaetae-substrate contact and by lifting the worm up off the substrate to reduce friction with the ventral surface of the body. Some species have chaetae that are modified into hook shapes; these are primarily tube-dwellers that use hooks to grip the inner walls of the tubes (Purschke et al., 2014; Merz, 2015), although some burrowing species have hooks as well (Figure 10B). Hooks have dentition of sizes that correspond with the distinct microstructure of the tubes made by that species (Merz, 2015), and some have partial coverings called “hoods” or “guards” whose function has not been studied but may be interpreted as ways to reduce friction when the hooks are not being used. Some chaetae are thick spines, some of which are used for cutting, e.g., in tube shortening when surrounding sediments erode (Nowell et al., 1989), but others have unknown functions (Rouse et al., 2022).

The parapodia that hold the chaetae also vary considerably among species, from larger structures that are often divided into two complex lobes, the upper notopodium and the lower neuropodium (Figure 10A), to so reduced they are barely visible (Figure 10B) (Rouse et al., 2022). Thicker, rigid rods within parapodia called acicula help maintain their shapes, increase their stiffness, and support the bundles of thinner chaetae that extend from the parapodia (Figure 10A). Comparison of the parapodial musculature of related species of scale worms that primarily swim *versus* crawl showed that the complex patterns of musculature were similar (with 21 different muscle groups identified) but that the muscles that support and move the chaetae were more numerous and thicker in crawlers (Allentoft-Larsen et al., 2021). Polychaetes with large parapodia and many chaetae are often those that live on top of sediments and crawl (but may also burrow); many worms that are primarily burrowers (e.g., earthworms and capitellids; Figure 10B) have reduced parapodia and fewer, shorter hairs (Purschke et al., 2014). This arrangement makes sense given the differences in forces involved in burrowing *versus* crawling; burrowers can expand their bodies to push on either dorsal and ventral walls of crack-shaped burrows or radial walls of cylindrical burrows, whereas crawlers have only one surface and normal forces depend on the weight of the animal (Dorgan, 2010).

Parapodia and chaetae vary considerably among annelids, and these variations are often used to identify species, yet understanding of how these variabilities translate to the locomotory performance of annelids is surprisingly poor. Direct visualization of how chaetae or even parapodia are used in burrowing is challenging because these structures are very small and are operated in an opaque environment. Merz and Edwards (1998) cut off the ends of jointed chaetae and measured decreases in performance of different locomotory gaits, showing that the jointed chaetae are important in traction, but these manipulative experiments on animals are very challenging. Development of robots with friction-enhancing mechanisms that mimic these biological differences could allow better understanding of the functions of these diverse structures.

Several surface innovations have been developed to provide robots with asymmetrical friction, mimicking the chaetae of annelid worms. Kirigami skins can include cutouts that protrude under pressure but lie flat when pressure is low, resulting in asymmetric friction (Zhang et al., 2021; Liu et al., 2019) (Figures 7E, 10D). Creating skins that enable forward and backward locomotion, with large variation in diameter, is a challenge that cannot be separated from the overall mechanical design of robots. Fabric skins can help hold the mechanical components together (Mehringer et al., 2017). Stretchable polymers have good potential for both large strains and impermeability, although there may be an inherent tradeoff between low stiffness for ease of actuation and durability which tends to increase stiffness.

## 6 Burrowing challenge 4: Sensing and navigation

Both burrowing animals and robots need to not only create space and move forward into it, but also to control the direction of motion, which includes maintaining a straight path either vertically or horizontally, turning to navigate around obstacles or toward targets, and receiving and processing the sensory cues needed for navigation. Turning is one of the main advantages of soft burrowing over rigid drills. Gradual turning is needed to correct any errors in path direction. Responsive turning is needed to get around impenetrable objects or areas that are not to be disturbed.

### 6.1 Steering

A burrower’s most immediate responses will be to the local environment. Since perception range may be more limited in soils and sediments, animals rely on the their tapered heads, which often include antennae, to explore and evaluate heterogeneous environments (Figure 2). Plant roots exhibit semi-circular rotations called circumnutations that have been demonstrated to reduce penetration resistance (Martinez et al., 2021). Among annelids, anterior sensory structures vary considerably in number, position, and length, but how they are used, e.g., in burrowing and navigation, has not been well studied (Jumars et al., 2015). However, probing with the head while holding the posterior in place could save energy as a worm determines where to go.

Worm bodies are challenging to turn, even on smooth surfaces. Along a straight path there is a symmetry of paired extending and retracting segments: the extending segments at the beginning are shaped like the retracting segments at the end of a phase of actuation (Kandhari et al., 2019a). If the robot is moving along an arc with constant curvature, the required segment shapes to keep the anchors in place are different for each segment in the body and for each wave. Softness of the body means that passive compliance may be able to meet these demands without excessively precise calculation, but this does create a non-holonomic body constraint. A non-holonomic constraint is one that cannot be calculated *a priori*, but is rather based on the current state of the body. The most common example is a wheel. It cannot move sideways; therefore the direction it can move is dependent on the angle of the wheel, which can change based on history of the system. In contrast, while a wheel or sailboat can be described with a single state variable (angle), the body of a worm will have many segments each with their own state variables. Algorithms are still being developed to take advantage of the full degrees of freedom of worms. Current approaches can search through trees of possible behaviors (Wang et al., 2020) and it is possible to follow smooth, especially Bezier curves to get to desired positions (Wang et al., 2021). Interestingly, in simulation, attempts to turn sharply result in curves in the body shape that persist over many cycles as the path straightens.

Within a burrow, steering is likely to be especially difficult. The mechanism of burrow extension by fracture provides some insight into how animals move forward on small scales: cracks propagate in the direction of least resistance, indicating that burrows may follow previous cracks or regions in which sediments are less compacted (Dorgan et al., 2006). A recent study uses finite element modeling of fracture and visualization of crack paths made by worms in natural sediments to suggest that worms are unable to steer crack path direction by pushing on one side of the burrow; rather, cracks branch when sediment heterogeneity is sufficient, and worms follow one branch or the other to change directions (Dorgan and Arwade, in review).

Furthermore, robots have had challenges in maintaining a desired depth. First, entering the soil can be difficult because above the soil there are weaker anchoring forces available for penetration. Sometimes a starting tube is used to brace the body as it enters (Omori et al., 2013). Secondly, burrowing horizontally in shallow soils means that there is greater resistance below than above, which causes the robot to tend to come up and out of the soil. Terra foils have helped with this problem (Drotman et al., 2022). Because fracture toughness increases with depth in natural sediments (Johnson et al., 2012), a crack propagating at a shallow angle is likely to be passively steered upward, whereas a crack propagating straight down is likely to follow a straight path.

Because soil properties change with pressure, it makes sense to use depth as part of the navigation strategy, which is what worms seem to do. Statocysts have been described in many different burrowers; these gravity-sensing organs comprise a dense statolith (in some cases, a sand grain) in a chamber lined by ciliated cells (Locke, 2000). The high density of soils and sediments could potentially allow estimation of depth from the overlying weight, although whether animals can detect this overburden has not been explored.

Thus, in muddy sediments in which animals or robots are burrowing by fracture, it seems that a good strategy is to initially burrow down at a shallow angle in which penetration resistance is low. Once the animal is deep enough to use the resistance of the sediment, then it turns and burrows straight down to the desired depth. This strategy avoids horizontal burrowing at depth, which would result in the burrow being steered upward. Marine burrowing animals need to maintain a burrow opening with the surface to irrigate their burrows with oxygenated water, so are unlikely to burrow horizontally over any substantial distance. Further biological experiments on how worms enter sediments, how they execute downward turns, and what cues affect burrow direction, e.g., when to burrow in non-vertical directions, will be interesting to engineers designing worm robots.

Some species of worms demonstrate other characteristic burrow shapes, for example, the J-shaped burrow of lugworms that create a vertical tail shaft that curves horizontally, enabling the worm to feed at a constant depth in different areas without having to create an entirely new burrow. Ingestion of sediments results in a collapse of surface sediments, creating a feeding funnel; the J-shaped burrow allows the worm to feed on sediments far enough from the fecal mound at the tail shaft to prevent re-ingestion (Figure 3B). Perhaps more impressive are the spiral-shaped burrows of polychaetes from the family Paraonidae, which are created along horizontal planes ∼ 5–10 cm below the sediment surface (Risk and Tunnicliffe, 1978). These and other similar burrows preserved in the fossil record conform to a simple algorithm for feeding in a patch of enriched food, in this case concentrated algae: keep turning to stay within the patch, avoid crossing paths where the animal has already fed, and stay in close proximity to previous tracks (Jumars, 1993). Spiral burrows are evidence of precise control over burrower movements within sediments, although the mechanisms are not well understood.

### 6.2 Mechanical sensing

Mechanosensing is likely another important mechanism used by burrowers, although it has been very understudied. Perhaps the most notable example is the effectiveness of worm grunting, in which a wooden stake is driven into the soil and a steel rod is rubbed against the wood to produce vibrations, resulting in emergence of earthworms from the soil, in this case to be collected and used as bait. These vibrations are similar to those produced by moles foraging for earthworm prey, and humans as well as wood turtles and herring gulls have figured out how to mimic the vibrations of these more common earthworm predators (Catania, 2008). In marine sediments, pressure signals generated by burrowing animals have been measured with pressure sensors up to 20 cm away from burrows that are ∼1 cm in diameter (Wethey and Woodin, 2005). It follows that these signals may also be detected by animals, although the implications of this in marine environments have yet to be explored. How worms may detect pressure signals has not been well studied, although the growing field of vibrational communication has provided insight on how diverse animals, primarily arthropods and mammals, use vibrations through substrate for communication (Hill, 2008). Polychaetes can have a variety of antennae and elongated appendages called cirri on their heads, tails, and along their bodies, as well as sensory cells with hairs that are likely mechanosensory but have not been studied experimentally (Purschke, 2005). Rigid objects may be detectable through increases in sediment stiffness that affect the resistance of muds to forces applied by worms, although this source of anisotropy has also not been explored. Worms burrowing in muds tend to preferentially burrow against rigid walls, whereas worms burrowing in sands avoid walls, presumably driven by the different mechanics of burrowing in these different media (Du Clos et al., 2013). The septa separating segments may allow separate detection of pressure as well as generation of pressure in different areas of the body.

For robots, an even more basic application of mechanical sensing can be in adapting their gaits to their substrates. A so-called “open-loop” gait, in which the amplitude of the peristaltic wave is constant is less efficient in variable-diameter burrows than if the robot can modulate the amplitude and timing of peristalsis (Daltorio et al., 2013). Worm robots can have embedded sensors for contact pressure and strain that can be used to estimate forward progress and slip (Wang et al., in revision) better than inertial measurements. This information could be used to evaluate whether the peristaltic gait needs to be altered for the environment or whether the robot is stuck and needs to back up. Soft robot designs will need to increasingly incorporate embedded sensing in order to navigate challenging confined environments.

### 6.3 Outlook for navigation

A basic behavior required for mobile robots is the ability to follow a predefined path with desired waypoints in space. Burrowing robots will need new strategies for estimating their locations in space, so that they can determine achievable paths to the waypoints. Furthermore, they will need to use varied approaches on different substrates; for example Du et al. (2022) propose a hybrid robot with different gaits for confined tubes *versus* flat surfaces. Modeling of soft interfaces between robots and their environments will require development, as has been done for growing robots (Blumenschein et al., 2020).

Non-etheless, live worms give hope for potentially reactive burrowing around important subsurface features, such as infrastructure. Additional understanding for how they plan their paths, avoid obstacles, and brace against available surfaces will inspire new robot behaviors. The challenges of worm-like locomotion will inspire new ways of developing smarter locomotion algorithms. Worm robots are good platforms because the animals are considered simple enough to duplicate, but control solutions will need to scale for many segments and react to a large number of distributed inputs.

## 7 Conclusion

Robotics and biology have complementary contributions to understanding peristalsitic locomotion. In biology, there is wealth of examples of organisms that live in environments where machines cannot yet go. The diversity of these animals provides positive proofs of new design possibilities. Biological experiments require ingenuity to develop methods to visualize and measure key parameters, but then can be performed on many different species. Engineering work can test resulting hypotheses about the minimum requirements for locomotion and effects of design on burrowing performance. It can compare alternate designs while removing behavioral variability. Yet, despite these commonalities there is a large gap in biological and engineering understanding of worm locomotion.

This review aims to bridge this gap by distilling the general problem of burrowing into four challenges, and considering how animals and robots address each. These challenges are making space, moving forward with a soft body, anchoring, and sensing and navigation. Some overall differences stand out:

Animals have adapted some of the same tactics for multiple challenges. For example, radial expansion helps make space *via* fracture, but also anchors. This confluence is no coincidence, animals find niches by using the skills they have. Helping robots learn to become multifunctional ties into the larger field of embodied intelligence.

Burrowing robots tend to be much larger than burrowing animals. Scaling appears to be most important in addressing the challenge of creating space; animals of different sizes use different mechanics to extend their burrows and have different morphologies to achieve it. Development of smaller soft robots is becoming more feasible with advances in soft robotics. Peristaltic robots have already provided new insight into how peristalsis works in animals, and small, burrowing peristaltic robots have great potential to address fundamental biological questions. Alternately, larger burrowing animals that excavate or fluidize sediments could provide inspiration for burrowing robots on those scales.

A constraint for engineered devices is the tethered connection. Robot burrowers are physically connected to the exterior of the burrow for power, pressurized fluid, control signals from the human user, or discharge ports. Robots can creatively use this connection—fluidizing sand, or growing with more material, or for allowing easy extraction without losing the robot. Potential exists for new tethering combinations, e.g., for a larger robot to deploy a smaller probe. At the same time, worms can renew appreciation for distributed and finely controlled biological muscle and structural tissue.

A desired behavior for robots is long, straight horizonal burrowing—but biological worms do not seem to do this in compacted soil. The engineering demand relates to infrastructure like cable installation or agriculture in which plants may be aligned in rows. This is an opportunity for engineering innovation (e.g., augers or terra foils) and also a reason to avoid horizontal drilling when possible. This is also a particular behavior for biologists to note if observed in one of the many under-studied marine worm species.

In both biology and robotics, the amount of sensory response and active *versus* passive control required for various tasks seems largely unknown. Developing this understanding is essential for both adaptability and scaling, but is challenging for both roboticists and biologists. Collaborative research in this area could lead to significant advances in both disciplines.
